# Smoking Cessation Apps for People with Schizophrenia: How Feasible Are m-Health Approaches?

**DOI:** 10.3390/bs12080265

**Published:** 2022-08-01

**Authors:** Chelsea Sawyer, Lamiece Hassan, Daniel Guinart, Luis Martinez Agulleiro, Joseph Firth

**Affiliations:** 1 Division of Psychology and Mental Health, The University of Manchester, Manchester Academic Health Science Centre, Manchester M13 9PL, UK; chelsea.sawyer@manchester.ac.uk (C.S.); lamiece.hassan@manchester.ac.uk (L.H.); 2 Institut Hospital del Mar d’Investigacions Mèdiques (IMIM), Institut de Neuropsiquiatria i Addiccions (INAD), Hospital del Mar, CIBERSAM, 08003 Barcelona, Spain; daniguinart@gmail.com; 3 Institute for Behavioral Science, Feinstein Institutes for Medical Research, Department of Psychiatry, Zucker School of Medicine at Northwell/Hofstra, New York, NY 11549, USA; 4 Psychiatry Department, Complexo Hospitalario Universitario de Ferrol, 15405 Ferrol, Spain; luis.miguel.agulleiro@gmail.com; 5 Greater Manchester Mental Health NHS Foundation Trust, Manchester Academic Health Science Centre, Manchester M13 9PL, UK

**Keywords:** smoking, m-Health, schizophrenia, behaviour change

## Abstract

**Background:** The large health disparities among those diagnosed with schizophrenia urgently need to be addressed. These disparities are partially caused by adverse health behaviours such as smoking. Smoking cessation apps vary in efficacy across various populations, and there are concerns regarding the accessibility and usability of apps for people with schizophrenia. **Objective:** This review identifies and examines the feasibility of using apps for smoking cessation in people with schizophrenia. **Methods:** A non-systematic narrative literature review of smoking cessation apps for individuals with schizophrenia was performed. **Results:** Eight studies were included in this review. **Conclusion:** Smoking cessation apps can be acceptable and feasible, but may need to be tailored to the needs of people with schizophrenia. **Key messages:** (1) Smoking cessation apps could be acceptable and feasible for use in people with schizophrenia; (2) Lack of motivation was perceived as the main potential barrier with regard to people with schizophrenia engaging with smoking cessation apps; (3) In order to improve motivation of people diagnosed with schizophrenia, apps could include games, rewards, and/or social support; (4) Smoking cessation apps with a simple interface seem to be beneficial for this population; (5) Apps may need to be tailored to consider this population’s mental health needs.

## 1. Background

People diagnosed with schizophrenia have large disparities in physical health when compared with the general population, as demonstrated by the ~15 years reduced life expectancy and increased risk of metabolic syndrome and cardiovascular disorders [1]. These disparities are partially due to side effects of antipsychotic drugs and reduced provision of adequate physical healthcare, alongside adverse health behaviours such as physical inactivity, poor diet and smoking.

Considerable attention has been devoted to various health behaviours interventions in schizophrenia, with extensive research on diet [2,3,4,5], physical activity [2,3,6,7,8], and smoking [9,10,11] in this population. Currently, however, health behaviour interventions are now increasingly looking to digital modes of delivery, including smartphone applications, ‘apps’, wearable devices and web-based programs, as harnessing these new technologies could, theoretically, maximise cost-effectiveness and widespread dissemination of behavioural interventions.

While the use of digital health interventions in schizophrenia remains under-researched overall, smoking cessation appears to have gained substantial research interest –with numerous studies already investigating the feasibility and acceptability of using smoking cessation apps in this population. This increased attention is arguably warranted given that people with schizophrenia are more likely to be heavy smokers than the general population, but also more likely to die as a result of tobacco use [12]. Strikingly, in the United States approximately half of all recorded smoking-related premature deaths are attributable to people with severe mental illness (SMI) [13]. Further, around 70% of all deaths are due to physical health conditions such as cardiovascular disease, cancer, and respiratory disease [14]. While motivated to quit smoking, people with schizophrenia face additional barriers to cessation, for example, smoking to cope/manage their mental health symptoms (improving cognitive dysfunction) and side effects [13,15], reducing their confidence in their ability to quit. Therefore, interventions designed for the general population may not be as effective or suitable for this particular group [14,15,16,17]. Additionally, concerns with the accessibility and usability of digital health interventions for SMI populations have been raised [16,17,18,19].

This non-systematic narrative review aims to summarise the existing literature on interventions using smoking cessation apps for individuals with schizophrenia. To do this, evidence from studies in mixed-diagnostic samples of people with schizophrenia in psychiatric care settings was considered. Specifically, this review sought to (i) identify digital smoking cessation techniques that have been used in people with schizophrenia, (ii) examine the feasibility of using apps for smoking cessation in people with schizophrenia, and (iii) explore the potential effects on smoking-related behaviours and mental health.

## 2. Methods

To identify the relevant studies for this non-systematic narrative review, literature searches were conducted in January 2022 using keyword search algorithms containing terms relevant to severe mental illness, health behaviour change and digital technologies. The searches were conducted of multiple academic databases, including the Cochrane Central Register of Controlled Trials along with the Health Technology Assessment, Allied and Complementary Medicine, APA PsycInfo, Embase, and MEDLINE(R) databases. Additional searches of Google Scholar and relevant publications’ citation lists were also conducted.

From the search results, English language articles reporting on any experimental/interventional studies examining the feasibility, usability or effects of digital smoking cessation in SMI populations were sought out. This included randomised control trials (RCTs), non-RCTs, quasi-experimental studies, and qualitative research. Of all the relevant studies included in this review, critical information on the studies’ sample (including size, mean age, diagnoses and demographics), intervention (including the delivery platform, length of intervention, control/comparator conditions) and findings (be it usage data, outcomes/effects or qualitative feedback) were extracted. Studies were excluded if the mobile component of the intervention only consisted of texts, emails or phone calls. These results are presented in Table 1 and synthesised narratively below.

## 3. Results

### Overall Studies and Type of Apps Used

Our study identified 15 potential papers; of those, five interventions were delivered online [20,21,22,23,24], one detailed the development of an app [25], and one study only recruited people with mood disorders [26], so they were excluded from this review. The remaining eight studies [27,28,29,30,31,32,33,34] were included and focused on a variety of apps (Quitpal, Kick.it, QuitGuide, Learn To Quit (LTQ), and quitSTART). The key features of these app and apps interfaces, we could detect, are presented in Table 2 and Table 3, respectively.

Three studies compared the usability of two different smoking cessation apps (LTQ and QuitGuide) in a schizophrenia sample [30,31,33]. Two used the same sample [31,34]. Vilardaga et al. (2019) conducted two case studies with crossover AB interventions, in which participants completed both interventions once, and the order of the intervention (QuitGuide and LTQ) was randomly allocated [30]. The System Usability Scale [35] (SUS; see Appendix A), was used to rate the usability of the interventions, SUS scores range from 0 to 100 and an SUS score above 68 is considered above average. The SUS scores for the two apps were found above the standard cut-off, with QuitGuide slightly outperforming LTQ for Participant 1 (who was diagnosed with schizophrenia), but largely underperforming LTQ in Participant 2 (who was diagnosed as unspecified psychotic disorder) [30]. Interviews were conducted to examine user experiences. The main themes for the LTQ app were “high retention and comprehension of smoking cessation content”, “engagement through gamification”, “simple to use and understand”, and “positive emotional experience”. The main themes for QuitGuide were “access to app features was challenging”, “serious look and feel”, “poor consensus about app content”, and the “tracking and charts were perceived useful and desirable” [30].

Browne et al. (2021) and Vilardaga et al. (2020) randomised 62 participants to either LTQ (*n* = 33 people) or QuitGuide (*n* = 29) [31,34]. All participants also received a smartphone with a data plan and 8-weeks supply of Nicotine Replacement Therapy (NRT). Over the 16-week follow-up period, both apps led to a reduction in cigarettes smoked, although the mean reduction in cigarettes per day was higher for LTQ participants (12.3 vs. 5.9 cigarettes) [31,34]. Browne et al. [32] reported that, compared to Quitguide, LTQ was also used on more days (34.1 vs. 32.0 days), for more minutes per day (228.0 vs. 129.4), and had a greater total number of interactions (335.5 vs. 212.7).

Additional randomized control trial (RCT) data presented in Vilardaga et al. (2020) reported that 12% of LTQ participants had biochemically verified 30-day point prevalence abstinence (PPA) from smoking, versus 3% of QuitGuide participants at follow-up [31]. LTQ participant app interaction was significantly longer overall (4.2 vs. 2.1 h; *p* = 0.044), and rated usability highly (SUS score: 85.2 vs. 78.4, *p* = 0.046) when compared to QuitGuide participants 29. A small reduction in anxiety, symptom severity, and depression at 4-month follow-up was found for the entire sample [31].

Two studies conducted by Gowarty et al. [28,33] compared the usability and effect on smoking of two apps: quitSTART and QuitGuide. Seventeen participants were randomly assigned to field-test one of the two apps independently for 2-weeks. While both apps have similar features, their design, content and target audience differ: QuitGuide was developed for adults, whereas quitSTART was developed for teenagers and included prompts to record quit attempts.

Both studies found initially that participants using QuitGuide scored overall satisfaction more highly, as measured via SUS, and task completion was slightly higher, with all 9 tasks being completed by 75% of participants, whereas 75% of quitSTART participants completed 8/9 tasks. However, satisfaction and ease of use ratings for quitSTART increased to match QuitGuide at the 2-week follow-up. The interviews supported the notion that after familiarisation with quitSTAR, the app was perceived as easy to use despite the initial navigation difficulties. Further, at follow-up, quitSTART was used for a greater number of days, had a greater number of interactions, and users responded to more notifications, when compared with QuitGuide. Overall, 78% and 75% of QuitGuide and quitSTART users, respectively, self-reported quitting or reducing smoking at follow-up; abstinence was biochemically verified (with breath CO < 7 ppm) in 25% of all quitSTART users [28,33].

Three other studies focused on a single smoking cessation app. The first, Klein et al. (2015), was qualitative and involved a two-stage interview process, with the themes from stage 1 informing the development of the second interview schedule [27]. The first stage interviews aimed to understand participants’ smoking-related experiences (smoking behaviour and history, effects of smoking on their mental health, motivation and attempts to quit, use of NRT). In this stage, participants reported that smoking helped them cope with mental illness and that their smoking increased when distressed. The second stage interviews explored experiences of using the Kick.it app (app features, content, functionality, aesthetics, and support). The key theme reported by this study was the importance of personalising the app to users’ psychosocial needs, specifically experiences of perceived stigma and social isolation among this population. Participants felt smoking helped them cope with mental illness and their smoking increased when distressed [27]. Therefore, apps could be adapted to consider mental health. Participants also felt that the app should normalise smoking relapses as part of the process of quitting, and that smoking cessation approaches should assist quitting smoking in real time, by providing techniques and support to reduce the urge to smoke when stressors arise in their daily lives. Some users found navigating the Kick.it app overwhelming, leading the authors to conclude that the app interface should be simplified. Finally, the tracking of cigarette use was perceived to be useful, and most participants (83%) were enthusiastic about engaging in a social network, favouring chatrooms to connect to others [27].

Vilardaga et al. (2016) conducted a small-scale (*n* = 5) usability study of the Quitpal app [32]. The mean SUS score (65.5) indicated usability below the industry standard. A reoccurring barrier found in the interviews was difficulty using the app due to multiple layers. Other themes were: smaller achievable goals, app focus should be reducing smoking, and the use of interactive and motivating features. Most users liked the reminder function [32].

Lastly, Wilson et al. (2019) conducted a mixed-methods study which aimed to refine an already existing smoking cessation intervention for people diagnosed with schizophrenia, schizoaffective disorder and psychotic disorder not otherwise specified [29]. The Multi-Component Mobile-enhanced Treatment for Smoking Cessation (iCOMMIT) consisted of (i) Stay Quit Coach app; (ii) a smartphone-based contingency management application (mCM); (iii) Cognitive Behavioural Therapy (CBT) counselling, which consisted of 5 sessions and a workbook, provided per cohort; and (iv) pharmacotherapy with bupropion, started 2 weeks before quit date. The study consisted of two consecutive cohorts, with five and eight participants, respectively. Changes were made to the app after the first cohort, which included removing the Stay Quit Coach app from the second cohort due to the low self-reported utilisation in the first. Most participants reported that the counselling sessions were the best part of the treatment package. Participants generally noted the mCM was acceptable, but some patients mentioned the difficulty of uploading while filming their CO readings [29].

## 4. Discussion

### 4.1. How Usable Are Smoking Cessation Apps?

Overall, studies demonstrated smoking cessation apps are feasible to use among people diagnosed with an SMI. When comparing overall SUS scores across smoking cessation apps, LTQ had an excellent SUS score (>80), while Quitpal [32], Quitstart [28,33], and in 2/3 studies using QuitGuide [28,33] SUS scores were below average (<70). Although impossible to directly compare with the other apps, it appears that the app designed specifically for patients with schizophrenia and other SMI (LTQ) was reportedly easier to use, possibly due to a simpler interface. Therefore, apps specifically developed and tailored for people with schizophrenia and other SMI may be more accessible and user friendly.

The need for tailoring apps to people’s mental health needs was a recurring theme across the interviews. Further, the individual’s technology skills (suggesting the simplification of app interfaces and ease of tracking behaviour), their symptomology and the impact that this has on their smoking, and the motivation to use the apps should be taken into consideration. To improve people’s motivation and increase engagement with the app, a variety of strategies were suggested, including prompts, social support, progress tracking, rewards (monetary, or virtual through badges), and/or gamification [27,33]. Furthermore, apps that were gamified and included badges and quizzes (quitSTART and LTQ), and had prompts (quitSTART) were associated with longer engagement times and a greater number of interactions when compared with non-gamified apps (such as QuitGuide) [26,28,29,31,32,33,34]. Hence, gamified apps with rewards such as badges, leader boards, etc., may be beneficial for this group to ensure initial engagement. However, all the studies had a relatively short follow-up, so the impact on sustained app engagement should be explored in future trials.

The desire for apps to have an element of social support, through either chat rooms or social media groups, with other users and/or smoking advisors was also highlighted, particularly when experiencing strong cravings or triggers [27,28,36]. Therefore, a smoking cessation app offering real-time social support and/or social support groups may be beneficial for people with schizophrenia. Alternatively, an app containing games, distraction techniques or quizzes could be useful if real-time support is not feasible.

### 4.2. Do Smoking Cessation Apps Reduce Smoking?

All the studies we included [28,31,33,34] found that smoking cessation apps led to a reduction in self-reported smoking. Two of the four studies found a significant reduction in smoking, determined by 30-day PPA, in patients assigned to LTQ when compared with QuitGuide (12% vs. 3%) [31,34]. The LTQ app also led to a significantly greater reduction in cigarettes compared with QuitGuide, although this was no longer significant when adjusted for the number of cigarettes smoked per day initially [31,34]. Additionally, participants assigned to QuitGuide had significantly more quit attempts (and thus, relapses) when compared with LTQ participants. Collectively, these findings may indicate an app designed specifically for those with schizophrenia and other SMI may be more favourable in this population.

The other two studies found that the proportion of participants who reported they had attempted to quit or reduce their smoking was similar for both apps (78% QuitGuide users vs. 75% quitSTART users) [28,33]. However, these studies only relied on self-reports, which are prone to recall bias, so it would be advisable that future studies use biochemical verification to confirm reduction in cigarette smoking. Due to the small sample sizes and short-term follow-ups, the effectiveness of smoking cessation apps cannot be fully determined, although it does appear that smoking cessation apps have great potential to help people with schizophrenia quit smoking, at least in the short term. Lastly, due to the narrative nature of this review, some smoking cessation apps already existing or in early development may have not been identified. Notwithstanding, this is, to our knowledge, the first comprehensive overview of the current state of affairs in the use of apps for smoking cessation in schizophrenia.

Participants reported that they were more likely to smoke when having worse mental illness symptoms and may be less likely to use an app at such times. However, none of the studies collected data regarding patient’s symptomology nor examined if it influenced app use. Further longitudinal RCTs are required to determine the long-term effectiveness of smoking cessation apps among those diagnosed with schizophrenia. Further work is needed to understand the impact of the patient’s symptomology on app use, their smoking cessation needs, and the effectiveness of the app. Such knowledge could be used to help people gain insight into their mental health and triggers for smoking relapse, and potentially identify additional support and coping mechanisms to manage their mental health.

## 5. Conclusions

Smoking cessation apps are feasible for people with schizophrenia. Apps may benefit from being tailored to the needs of people with schizophrenia, considering both layout (simple interface), content (normalising relapses and integrating symptomology and smoking), and components of the intervention (rewards, social support, etc.) to enhance engagement. There is a dearth of evidence to determine whether apps developed for those with schizophrenia and other SMIs are more effective at smoking cessation than apps designed for the general population. Additionally, there is an absence of evidence to determine if smoking cessation apps are effective at sustaining quit attempts long term. One limitation of these conclusions is the non-systematic narrative review methodology applied. Thus, further systematic reviews and meta-analyses on this topic might provide more definitive insights into the efficacy and utility of using digital devices for smoking cessation in mental healthcare.

## Figures and Tables

**Table 1:** Summary of each of the studies included in the review.

Authors, Country	Study Design, Length of Follow Up	Outcome Measures	Intervention Design	Apps Used		Sample		Population			
				App 1	App 2	Target Sample	Sample Size (N)	Mean Age (SD; Range)	Smartphone Ownership	Gender (male %)	Diagnosis
Browne et al., 2021, United States	Pilot RCT, 16 week	App Engagement measured by background analytics of app utilisation: (i) the number of interactions with app content (ii) minutes/day of app use (iii) number of days used. Smoking: Change in self-reported cigarettes per day from baseline to endpoin	8-weeks NRT Randomised to an app All received a smartphone with a data plan.	QuitGuide	Learn to Quit (LTQ)	(i) ≥18 years old (ii) ICD-10 diagnosis of an SMI (iii) Smoke ≥5 cigarettes daily	All = 62 LTQ = 33 QG = 29	LTQ 46.1 (SD 11.3) Quitguide 45.6 (SD 10.9)	Not included	LTQ 36.4% Quitguide 44.85%	Schizophrenia: 24.2% Bipolar disorder: 48.4% Major depressive disorder 27.4%
Gowarty Aschbrenner, & Brunette, 2021 United States	Usability study, 2-week field test of independent use	Usability measured using: (i) A semi-structured interview (ii) Usability and acceptability questionnaire	Randomised to an app	QuitGuide	quitSTART	(i) 18–35 years old (ii) Receiving treatment for SMI. (iii) A smartphone user.	17	29.0 (SD 3.9)	94.1% used smartphone ≥ twice daily.	58.8% entire sample	Psychotic disorder 41.1% SMI-PTSD 58.9%
Gowarty, Longacre, et al., 2021, United States	Usability study, 2-week field test of independent use	Usability measured using: (i) Video-recorded task completion protocol. (ii) Backend app use data, (iii) Open-ended interviews, (iv) Non-participant observation (v) Structured interviews (vi) SUS	Randomised to an app	QuitGuide	quitSTART	(i) 18–35 years old (ii) Receiving treatment for SMI. (iii) A smartphone user.	17	29.0 (SD 3.9)	94.1% used smartphone ≥ twice daily.	58.8% entire sample	Psychotic disorder 41.1% SMI-PTSD 58.9%
Klein et al., 2019, United States	Qualitative Study, No follow-up	2-stage interview process Stage 1: interviews to understand participants smoking related experiences. Stage 2: interviews explored smoking related experiences regarding the use of the Kick.it app	n/a	Kick.it		(i) ≥18 years old (ii) self-reported SMI diagnosis (iii) Attempted to quit smoking in the last 12 months or ex-smokers	12	Range: 31–53 Median: 47.5	75.0%	66.7%	Schizophrenia disorder 75.0%, Borderline personality 16.7% disorder, Bipolar disorder 8.3%
Vilardaga et al., 2016, United States	Usability study, <2 days experience with app	(i) Interviews (ii) task performances (iii) usage logs (iv) self-reported usability score	n/a	QuitPal		(i) ≥18 years old	5	51.2 (SD 4.3)	Not stated	100%	Schizophrenia 40.0% Schizoaffective 20.0% Bipolar disorder 20.0% Depression recurrent 20.0%
Vilardaga et al., 2019, United States	Usability study	Usability measured using: (i) User eXperience (UX), (ii) User Engagement (UE)	Randomised to an app	QuitGuide	Learn to Quit (LTQ)	(i) ICD-10 diagnosis of an SMI	7	45.0 (SD 9.5)	Not stated	42.9%	Schizophrenia 14.2% Psychotic disorder 28.6% Mood disorder 57.2%
Vilardaga et al., 2020, United States	Pilot RCT, 16 week	App Engagement Usability score Smoking: self-reported number of cigarettes smoked per day. Smoking abstinence was biochemically verified by a 7-day point prevalence abstinence.	Randomised to an app	QuitGuide	Learn to Quit (LTQ)	(i) ≥18 years old (ii) ICD-10 diagnosis of an SMI (iii) Smoke ≥5 cigarettes daily	62 LTQ = 33 QG = 29	LTQ 46.1 (SD 11.3) QuitGuide 45.6 (SD 10.9)	LTQ 83% QG 90%	LTQ 36.4% QG 44.8%	Recurrent major depression 27.4% Bipolar I or II 48.4% Schizophrenia spectrum 24.2%
Wilson et al., 2019, United States	Qualitative	Interviews were used to revisie the intervention Self-Reported Abstinence: timeline followback for tobacco use over the past 30 days (Sobell & Sobell, 1992). Self- reported abstinence was biochemically verified.	Two cohorts. Following the interviews from Cohort 1, revisions were made. The Multi-Component Mobile-enhanced Treatment for Smoking Cessation intervention consisted off: (i) Stay Quit Coach app * (ii) Smartphone-based application (mCM) (iii) Cognitive Behavioural Therapy Counselling: 5 sessions & workbook (iv) Pharmacotherapy: prescribed Bupropion and started prescription two weeks before quit date.	Stay Quit Coach	Smartphone-based application (mCM) participants uploaded video recordings of their CO readings to a secure website.	(i) Aged 18–70, (ii) smoke ≥ 10 cigarettes daily & smoking for > ≥ 1 year (iii) met criteria for schizophrenia, schizoaffective disorder or another psychotic disorder.	Cohort1 = 5 Cohort 2 = 8	47.8 (SD 11.0)	Not stated	Not stated	For both cohorts: Schizophrenia 53.8%% Schizoaffective disorder 38.5%, Psychotic disorder not specified 7.7%

* Removed for cohort. Abbreviations: CBT—Cognitive Behavioural Therapy; ICD—International Classification of Diseases; LTQ—Learn To Quit; MCM—Multi-Component Mobile-enhanced; NRT—Nicotine Replacement Therapy; PPA—Point Prevalence Abstinence; PTSD—Post-Traumatic Stress Disorder; QG—QuitGuide; RCT—Randomised Control Trial; SD—Standard Deviation; SMI—Severe Mental Illness; SUS—System Usability Scale.

**Table 2:** Summary of the features of the apps.

App	Target Population	Features on all	Additional Features (Explicitly Stated)											
			List Reason for Quitting	Daily Missions	Monitor NRT	Rewards/Incentives	Support from a Smoking Cessation Advisor	Presents Health Outcomes	Games/Distraction Tasks	Track Mood	Medication Plan & Prompt to Use NRT	Coping Plans & Breathing Exercises	Quiz	Social Support
Kick.it (adapted)	Adults	Set a quit date Monitor smoking Tips om coping with cravings Tips on relapses Review progress provide information about the consequences of smoking Prompts/notifications to use app presents financial benefits		X	X	X	X	X	X					X
Learn to Quit (LTQ)	People with an SMI			X	X	X		X	X	X			X	X
QuitGuide (QG)	Adults		X			X				X				X
Quitpal	Adults						X	X		X				
quitSTART	Teenagers		X			X			X	X				
Stay Quit coach *	Veterans		X		X		X	X			X	X		

Abbreviations: NRT—Nicotine Replacement Therapy; SMI—Severe Mental Illness; * Also provides information on PTSD and smoking.

**Table 3:** Summary of the user interface of the apps.

App	Target Population	Interface				Studies Which Used This App
		Text Only	Videos	Images/ICONS	5 or More Tabs	
Kick.it (adapted)	Adults	no	yes	yes	Not Presented *	Klein et al., 2019
Learn to Quit (LTQ)	People with an SMI	no	no but used sliding cartoons	yes	Yes	Browne et al., 2021; Vilardaga et al., 2019; Vilardaga et al., 2020
QuitGuide (QG)	Adults	no	no	Only the use of graphs and face emojis	Yes	Browne et al., 2021; Gowarty, Longacre, et al., 2021; Gowarty Aschbrenner, & et al., 2021; Vilardaga et al., 2019; Vilardaga et al., 2020
Quitpal	Adults	no	yes	yes	yes	Vilardaga et al., 2016
quitSTART	Teenagers	no	no	yes	yes	Gowarty, Longacre, et al., 2021; Gowarty Aschbrenner, et al., 2021
Stay Quit coach	Veterans	yes	no	no	No	Wilson et al., 2019

* No images presented in papers.

## Data Availability

Not applicable.

## Abbreviations

CBT	Cognitive Behavioural Therapy
ICD	International Classification of Diseases
iCOMMIT	Mobile-enhanced Treatment for Smoking Cessation
LTQ	Learn To Quit
MCM	Multi-Component Mobile-enhanced
NCI	National Cancer Institute
NRT	Nicotine Replacement Therapy
PPA	Point Prevalence Abstinence
PTSD	Post-Traumatic Stress Disorder
QG	QuitGuide
RCT	Randomised Control Trial
SD	Standard Deviation
SMI	Severe Mental Illness
SUS	System Usability Scale

## Appendix A. The System Usability Scale

Five-point Likert scale, ranging from Strongly disagree (1) to Strongly Agree (5)

(1) I think that I would like to use this system frequently.
(2) I found the system unnecessarily complex.
(3) I thought the system was easy to use.
(4) I think that I would need the support of a technical person to be able to use this system.
(5) I found the various functions in this system were well integrated.
(6) I thought there was too much inconsistency in this system.
(7) I would imagine that most people would learn to use this system very quickly.
(8) I found the system very cumbersome to use.
(9) I felt very confident using the system.
(10) I needed to learn a lot of things before I could get going with this system.
